# Delayed diagnosis of posterior cruciate ligament avulsion following lower limb fracture surgery: a case report

**DOI:** 10.1097/RC9.0000000000000576

**Published:** 2026-06-03

**Authors:** Saeed Kargar-Soleimanabad, Salman Ghaffari, Mehran Razavipour, Mostafa Kalteh

**Affiliations:** a Student Research Committee, Faculty of Medicine, Mazandaran University of Medical Sciences, Sari, Iran; b Faculty of Medicine, Orthopedic Research Center, Mazandaran University of Medical Science, Sari, Iran

**Keywords:** avulsion, posterior cruciate ligament, trauma

## Abstract

**Introduction:**

Avulsion fractures of the posterior cruciate ligament (PCL) are rare occurrences following knee trauma and are frequently missed during the initial assessment. These injuries are often overlooked due to subtle radiographic signs and the difficulty of performing a thorough physical examination in the presence of acute pain. Prompt and accurate diagnosis is essential to prevent long-term functional impairment of the knee.

**Case presentation:**

We report the case of a 42-year-old woman who sustained trauma to her left lower limb after falling from a height of approximately 3 m. She underwent orthopedic surgery for fractures in the left foot and was discharged in stable condition. However, during the second follow-up visit, persistent discomfort around the knee prompted further imaging. Radiographs revealed a previously undiagnosed avulsion fracture of the PCL in the left knee, which had not been identified during the initial trauma evaluation.

**Clinical discussion:**

This case highlights the diagnostic challenges associated with PCL avulsion fractures, particularly when overshadowed by more apparent injuries. It underscores the importance of maintaining a high index of suspicion and conducting comprehensive evaluations, including repeat imaging when clinical symptoms persist.

**Conclusion:**

Clinicians should be aware of the potential for missed PCL avulsion fractures, especially in polytrauma patients. Early recognition and management are critical to ensuring optimal functional recovery.

## Introduction

The posterior cruciate ligament (PCL) is one of the strongest intracapsular ligaments of the knee joint, originating from the anterolateral aspect of the medial femoral condyle and inserting into the posterior intercondylar area of the tibia. It acts as the primary posterior stabilizer of the knee, resisting posterior translation of the tibia relative to the femur and playing a critical role in maintaining knee joint stability^[^1,2^]^. PCL injuries are relatively uncommon compared to anterior cruciate ligament (ACL) injuries, accounting for approximately 3–20% of all knee ligament injuries^[^3^]^. Due to the ligament’s inherent strength, isolated PCL injuries are rare. When they do occur, they often involve avulsion of the ligament from its tibial attachment rather than mid-substance rupture^[^4,5^]^. PCL avulsion fractures, where the ligament pulls off a fragment of bone – typically from the tibial side – are more frequently observed in younger patients or in the setting of high-energy trauma, such as traffic accidents or falls from height^[^6^]^. These injuries may lead to posterior instability and, if left untreated, can result in chronic instability, functional impairment, and early-onset degenerative joint disease^[^7^]^.

HIGHLIGHTS

- The posterior cruciate ligament (PCL) is one of the strongest intracapsular ligaments in the knee joint.
- The PCL acts as the knee’s main posterior stabilizer.
- The PCL tibial avulsion can cause knee instability.

In polytrauma patients or those presenting with multiple fractures, the focus of initial evaluation is frequently directed toward obvious skeletal injuries. This diagnostic bias, combined with limited physical examination due to pain, immobilization, or prioritization of life-threatening injuries, can contribute to missed diagnoses of ligamentous injuries such as PCL avulsion^[^8^]^. Radiographic signs of avulsion may also be subtle or overlooked unless specifically sought through targeted imaging techniques like magnetic resonance imaging (MRI) or specific knee radiographs^[^9^]^. Delayed or missed diagnosis of PCL avulsion is not uncommon, particularly when initial radiologic evaluation is focused on fracture management. Such cases may only be identified during later follow-up when persistent knee pain, swelling, or instability prompt further investigation. Therefore, clinicians should maintain a high index of suspicion for occult ligamentous injuries in trauma patients, especially when clinical symptoms persist beyond the expected postoperative course^[^10^]^.

In this report, we present a rare case of a PCL tibial avulsion fracture that was not detected during the initial trauma workup or postoperative period but was diagnosed at the second follow-up visit following surgical treatment for lower limb fractures. This case underscores the importance of systematic re-evaluation, comprehensive imaging, and maintaining clinical vigilance for ligamentous injuries in the post-traumatic setting.

## Case presentation

**Figure 1.**
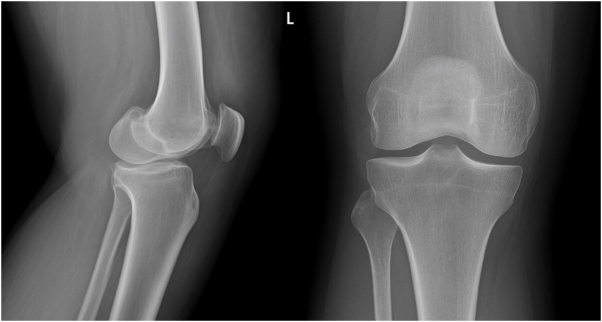
Legs’ front view and side view radiography immediately after trauma.

A 42-year-old woman was referred to an academic medical center following a fall from a height of approximately 3 m. Her medical history was significant for breast cancer, for which she had undergone breast mass resection 9 months earlier, followed by eight cycles of chemotherapy. The final chemotherapy session had been completed 1 month before the trauma. Upon arrival at the emergency department, the patient reported severe pain in her left lower extremity, which limited the initial physical examination. She was referred for radiologic assessment. Plain radiographs were obtained, including anteroposterior (AP) pelvic view; AP and lateral views of the left knee, leg, and ankle; and AP and lateral views of the left elbow, forearm, and wrist (Fig. 1).

Imaging revealed a distal radius fracture in the left upper limb and a tibia–fibula fracture in the left lower limb. A leg splint was applied, and the affected limb was elevated and subjected to cold compression. Preventive measures for compartment syndrome were initiated, and prophylactic anticoagulant therapy was started. Two days after admission, the patient underwent surgical intervention. The distal radius fracture was treated with percutaneous pinning and the application of an external fixator. For the tibia–fibula fracture, open reduction and internal fixation (ORIF) were performed using a 12-hole distal tibial anatomical plate, fixed with four screws in the proximal segment, four screws distally, and one cancellous screw. All procedures were performed under general anesthesia. The postoperative course was uneventful, and the patient was discharged in stable condition 2 days following surgery. A follow-up visit was scheduled for 10 days later.

**Figure 2.**
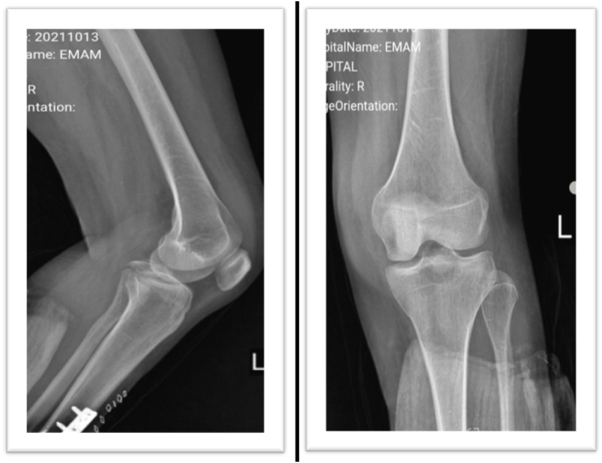
Legs’ front view and side view radiography after follow-up.

At the 10-day follow-up appointment, the patient reported persistent discomfort in the left knee. Repeat plain radiographs of the left knee (AP and lateral views) were obtained and revealed a PCL avulsion fracture, which had been missed during the initial evaluation (Fig. 2). Following identification of the PCL avulsion fracture at the second follow-up, the patient underwent structured conservative management, including hinged knee bracing, partial weight-bearing, and quadriceps-strengthening physiotherapy. Serial follow-ups demonstrated gradual improvement without instability. Surgical intervention was deemed unnecessary. This delayed diagnosis underscores the importance of comprehensive reassessment in polytrauma patients, especially when severe pain hinders complete physical examination at the time of admission. The current study has been reported in line with the SCARE criteria^[^11^]^.

## Discussion

PCL avulsion fractures are rare injuries, particularly in adult populations, and their diagnosis can be challenging, especially in the context of polytrauma, where clinical priorities are directed toward managing more apparent fractures. In the presented case, the delayed diagnosis of a PCL avulsion fracture until the second follow-up visit highlights several important considerations in trauma care, imaging strategy, and clinical vigilance.

Our patient sustained a high-energy fall from approximately 3 m, resulting in multiple fractures, including distal radius and tibia–fibula fractures. These injuries drew immediate clinical attention and appropriately required surgical fixation. However, the presence of significant lower extremity pain and immobilization limited the initial physical examination, likely contributing to the missed ligamentous injury, a pattern consistent with similar cases reported in the literature^[^7,12^]^.

In another study, a PCL avulsion fracture associated with a tibial plateau fracture was not detected until late follow-up, despite initial imaging. The authors emphasized that distraction by other skeletal injuries and inadequate imaging contributed to the delayed diagnosis^[^13^]^. Similarly, Raj *et al* found that early diagnosis of PCL avulsion requires targeted radiologic evaluation, which is often omitted when the patient presents with multiple injuries or where pain limits joint-specific testing^[^14^]^. These findings align with our case, where the initial imaging was comprehensive in scope but not focused specifically on intra-articular evaluation of the knee. Although PCL avulsion fractures are not exceedingly rare, this case illustrates an under-recognized diagnostic challenge in polytrauma settings, where pain, immobilization, and clinical prioritization frequently mask ligamentous injuries. Highlighting this scenario reinforces the importance of systematic re-evaluation and repeat imaging when symptoms persist.

Biomechanically, the PCL is a robust ligament, and avulsion injuries typically occur due to hyperflexion or a direct blow to the proximal tibia while the knee is flexed – mechanisms that are plausible in high-impact trauma, such as falls from a height^[^15,16^]^. In our case, the force of impact likely resulted in concurrent bony and ligamentous trauma; however, the clinical presentation was dominated by the more severe tibia–fibula fracture, leading to diagnostic overshadowing of the intra-articular injury. At the first postoperative follow-up (day 10), the patient continued to experience knee discomfort disproportionate to the expected postoperative pain trajectory. This prompted repeat imaging, which revealed the avulsed PCL fragment. This decision to reassess and investigate persistent symptoms was critical. In contrast, delayed or unrecognized PCL injuries – especially avulsion types – can result in chronic posterior instability, altered joint mechanics, and early degenerative changes if not treated appropriately^[^17^]^. Unlike some reported cases where delayed diagnoses necessitated complex reconstructions or secondary surgeries, our case allowed for early identification during the subacute phase, creating an opportunity for more favorable outcomes. This reflects best practice in trauma management: any persistence of symptoms beyond the expected postoperative course warrants a systematic re-evaluation, both clinically and radiographically. From an educational perspective, this case underscores the value of maintaining a high index of suspicion for ligamentous injuries in patients with polytrauma. It also emphasizes that the absence of findings on initial imaging – especially when not knee-specific – should not preclude further investigation when clinical symptoms persist. As suggested by Naraghi *et al*, imaging strategies for knee trauma should incorporate MRI or high-quality dedicated radiographs when physical examination is unreliable^[^18^]^.

## Conclusion

The PCL avulsion might occur following knee trauma and in the absence of a thorough examination and complementary imaging. Therefore, it is necessary to examine this case with great accuracy in knee trauma.

## Data Availability

The datasets used and analyzed during the current study are available from the corresponding author on reasonable request.

## References

[1] LaPrade RF Moulton SG Nitri M. Clinically relevant anatomy and what anatomic reconstruction means. Knee Surg Sports Traumatol Arthrosc 2015;23:2950–59. 25957611 10.1007/s00167-015-3629-1

[2] Race A Amis AA. The mechanical properties of the two bundles of the human posterior cruciate ligament. J Biomech 1994;27:13–24. 8106532 10.1016/0021-9290(94)90028-0

[3] Mook WR Miller MD Diduch DR. Multiple-ligament knee injuries: a systematic review of the timing of operative intervention and postoperative rehabilitation. J Bone Joint Surg Am 2009;91:2946–57. 19952260 10.2106/JBJS.H.01328

[4] Jang K-M Lee S-H. Delayed surgical treatment for tibial avulsion fracture of the posterior cruciate ligament in children. Knee Surg Sports Traumatol Arthrosc 2016;24:754–59. 26704790 10.1007/s00167-015-3929-5

[5] Mishra AK Vikas R. A rare case of bony avulsion of posterior cruciate ligament from its femoral attachment. Med J Armed Forces India 2016;72:S98–s100. 28050083 10.1016/j.mjafi.2015.11.014 PMC5192177

[6] Ghilley SK Sansi NK Meena M. Outcome of isolated PCL tibial avulsion fractures treated with cannulated cancellous screw fixation. J Orthop Spine Trauma. 2022;3:15–19.

[7] Shelbourne KD Davis TJ Patel DV. The natural history of acute, isolated, nonoperatively treated posterior cruciate ligament injuries. A prospective study. Am J Sports Med 1999;27:276–83. 10352760 10.1177/03635465990270030201

[8] Takahashi T Watanabe S Ito T. Current and future of anterior cruciate ligament reconstruction techniques. World J Meta-Anal 2021;9:411–37.

[9] Helito PV Peters B Helito CP. Imaging evaluation of the multiligament injured knee. Ann Joint 2018;3:UNSP80.

[10] McCoy JS Fractures NRA. StatPearls. Treasure Island (FL): StatPearls Publishing Copyright © 2025, StatPearls Publishing LLC; 2025.

[11] Kerwan A Al-jabir A Mathew G. Revised surgical CAse REport (SCARE) guideline: an update for the age of artificial intelligence. Prem J Sci 2025;10:2025.

[12] Guimarães TM Helito PVP Angelini FJ. Delayed treatment of a posterior cruciate ligament tibial insertion avulsion fracture in a child with open physis: a case report with a 4-year follow-up. J Pediatr Orthop B 2017;26:477–81. 28742679 10.1097/BPB.0000000000000432

[13] Gwinner C Kopf S Hoburg A. Arthroscopic treatment of acute tibial avulsion fracture of the posterior cruciate ligament using the tightrope fixation device. Arthrosc Tech 2014;3:e377–e82. 25126507 10.1016/j.eats.2014.02.005 PMC4130139

[14] Raj MA Mabrouk A Varacallo MA. Posterior cruciate ligament knee injuries. StatPearls. StatPearls Publishing; 2023. 28613477

[15] Fanelli GC Edson CJ. Posterior cruciate ligament injuries in trauma patients: part II. Arthroscopy 1995;11:526–29. 8534292 10.1016/0749-8063(95)90127-2

[16] Cho YT Lee JH Yoon JH. Knee morphology and proximal tibial bone quality around the posterior cruciate ligament insertion site affect injury patterns. Clin Orthop Surg 2025;17:400–07. 40454134 10.4055/cios24440 PMC12104027

[17] Lee BK Nam SW. Rupture of posterior cruciate ligament: diagnosis and treatment principles. Knee Surg Relat Res 2011;23:135–41. 22570824 10.5792/ksrr.2011.23.3.135 PMC3341837

[18] Naraghi AM White LM. Imaging of athletic injuries of knee ligaments and menisci: sports imaging series. Radiology 2016;281:23–40. 27643766 10.1148/radiol.2016152320

